# Effect of short-term ischemia on microcirculation and wound healing of adipocutaneous flaps in the rat ^1^

**DOI:** 10.1590/s0102-865020190120000003

**Published:** 2020-02-10

**Authors:** Abel Molnar, Zsuzsanna Magyar, David Belkin Nachmias, Din Mann, Balazs Szabo, Laszlo Toth, Norbert Nemeth

**Affiliations:** I MD, Department of Operative Techniques and Surgical Research, Institute of Surgery, Faculty of Medicine, University of Debrecen, Hungary. Technical procedures, analysis and interpretation of data, manuscript preparation.; II PhD, Department of Operative Techniques and Surgical Research, Institute of Surgery, Faculty of Medicine, University of Debrecen, Hungary. Technical procedures, analysis and interpretation of data, manuscript preparation.; III MD, Department of Operative Techniques and Surgical Research, Institute of Surgery, Faculty of Medicine, University of Debrecen, Hungary. Technical procedures, measurements, acquisition of data.; IV MD, Department of Operative Techniques and Surgical Research, Institute of Surgery, Faculty of Medicine, University of Debrecen, Hungary. Manuscript preparation.; V PhD, Department of Pathology, Faculty of Medicine, University of Debrecen, Hungary. Histopathological examinations.; VI PhD, DSc, Department of Operative Techniques and Surgical Research, Institute of Surgery, Faculty of Medicine, University of Debrecen, Hungary. Conception and design of the study, analysis and interpretation of data, manuscript preparation.

**Keywords:** Ischemia, Reperfusion, Microcirculation, Wound Healing, Histology, Rats

## Abstract

**Purpose:**

Composite flaps used in reconstructive surgery may intra- and postoperatively suffer from hypoperfusion and/or ischemia-reperfusion influencing wound healing. We aimed to follow-up the effect of ischemia on adipocutaneous flaps’ wound healing and microcirculation.

**Methods:**

In anesthetized rats groin flaps were formed bilaterally. In Control group the flaps were repositioned and sutured back. In Ischemia-Reperfusion (I/R) group before repositioning and suturing the flap pedicles were clamped for 60 minutes. Laser Doppler (LD) fluxmetry and temperature probes were applied on the cranial, central and caudal flap regions before/after preparation and ischemia, re-suturing, and on the 1^st^-3^rd^-5^th^-7^th^-14^th^ postoperative days, before the final examinations and biopsies for histology.

**Results:**

Flaps’ skin temperature quickly recovered after repositioning. LD values were lower in the I/R group, reaching a significant level by the 3^rd^ postoperative day, and remained lowered till the 14^th^ day. The magnitude of alterations differed in the flap regions. Histologically normal wound healing process was seen, except for some I/R flaps, where hypertrophized mammary glands were found.

**Conclusions:**

Short-term ischemia could influence flap microcirculation and wound healing, and may result in hypertrophized mammary glands. Laser Doppler could be used to evaluate intra- and postoperative microcirculatory changes and may have significance in predicting complications.

## Introduction

Viability of various composite flaps is a key factor of the successful reconstructive operation. Numerous clinical and experimental studies demonstrated that microcirculatory disturbance may lead to flap failure^1 - 4^ . Mechanical damage during flap preparation and positioning, bending and torquation of pedicle’s vessels, thrombosis, edema, inflammation, all may lead to complication, resulting in flap necrosis^5 , 6^ . However, several postoperative complications cannot be predicted easily. Intra- and postoperative monitoring of flap microcirculation may provide useful information for better understanding and following-up the ischemia-reperfusion-related alterations in tissue perfusion^6 - 10^ .

Causes for flap ischemia and necrosis include: (1) preoperative ones, such as inadequate flap design and sizing, damaging flap’s blood supply, previously radiated, operated or traumatized tissue, and risk from the patient’s side (e.g., smoking, hypertension, etc), (2) intraoperative causes jeopardizing the flap tissues and its vasculature leading to early venous congestions, venous or arterial thrombosis, and (3) postoperative ones: hematoma, thrombosis, infection^11^ . Many of these factors can be investigated well in experimental conditions, revealing better the pathophysiological background.

Numerous microsurgical flap models are known used in rodents with various tissue composition and requiring different level of technical skills^13 , 14^ . Groin flap model is a feasible model to study adipocutaneous skin flap physiology and pathophysiology^14 , 15^ . However, the ischemic time in the models is different. Although the skin has a relatively wide ischemic tolerance, the border or reversible and irreversible alterations are still unknown. Investigating this issue, various techniques for monitoring flap perfusion and microcirculation provide useful data^12 , 16 - 23^ .

We hypothesized that even a short-term ischemia may influence flaps’ wound healing, since the endothelium itself, existing in all tissues, is very sensitive for hypoxia^9 , 24 , 25^ . We also hypothesized that microcirculatory blood flux units differ at various sites of the flap and may predict the postoperative complications. In this study we aimed to investigate and follow-up the effect of a short-term ischemia and the following reperfusion on adipocutaneous flaps’ microcirculation and wound healing.

## Methods

### Experimental animal, operative techniques and investigative protocol

The experiments were approved and registered by the University of Debrecen Committee of Animal Welfare (permission registration Nr.: 20/2011. UDCAW), in accordance with the relevant Hungarian Animal Protection Act (Law XVIII/1998). Seventeen male Crl:WI outbred rats (Toxi-Coop Ltd., Hungary; body-weight: 399.5±70.7g) were anesthetized with sodium-thiopental (60 mg/kg, i.p.).

**Figure 1:**
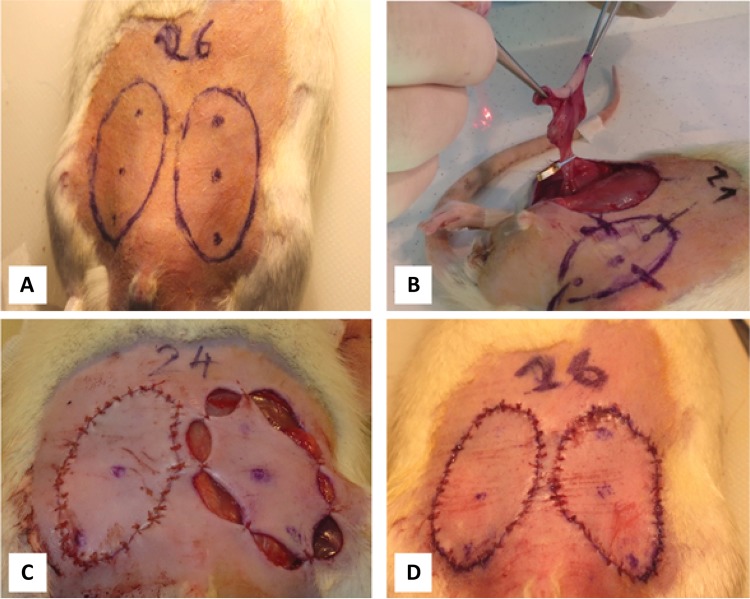
Main steps of the experimental operations. A: marking bilaterally the groin flap incisions using a template, defining the laser Doppler measurements sites (cranial, central and caudal regions), B: after skin incision gentle preparation and elevation of the flap, and clamping of the pedicle (superficial inferior epigastric vessels), C: skin stitches, carefully balancing the stretching, D: sutured flaps.

Adipocutaneous groin flaps were prepared bilaterally, using an ellipsoid template, whose area was 8.24 cm^2^. The skin incision line was pre-marked, also indicating the measurement points for temperature and microcirculation at the cranial, central and caudal region of the flaps. In the Control group (n=10) after 1 hour we repositioned and sutured the flaps with 32 stitches (4/0, Dexon), taking care of tension-free suturing. In the Ischemia-Reperfusion group (I/R, n=7) microvascular clips were placed on the flaps’ pedicle containing the superficial inferior epigastric vessels. After 60 minutes of ischemia the clips were removed, and the flaps were repositioned and sutured as described above ( Fig. 1, A-D ).

Skin surface temperature and microcirculatory measurements were performed on the cranial, central and caudal flap regions before the skin incision (base), after flap preparation, by the end of ischemia (I-60/R-0), 5 minutes after removal of clips (R-5), after re-suturing, and on the 1^st^, 3^rd^, 5^th^, 7^th^and 14^th^ postoperative days.

Besides daily wound control, Flunixin (2 mg/kg, s.c.) was administered on the 1^st^, 3^rd^, 5^th^ postoperative days. To avoid autophagy, we used a plastic collar on the first few postoperative days. In case of suture insufficiency debridement and suture replacement were performed under anesthesia. Skin surface temperature and microcirculatory measurements were also performed on the 1^st^, 3^rd^, 5^th^, 7^th^ and 14^th^ postoperative days. On the 14^th^ p.o. day in anesthesia, the flaps were excised for histological examinations.

### Skin surface temperature and microcirculatory measurements

**Figure 2:**
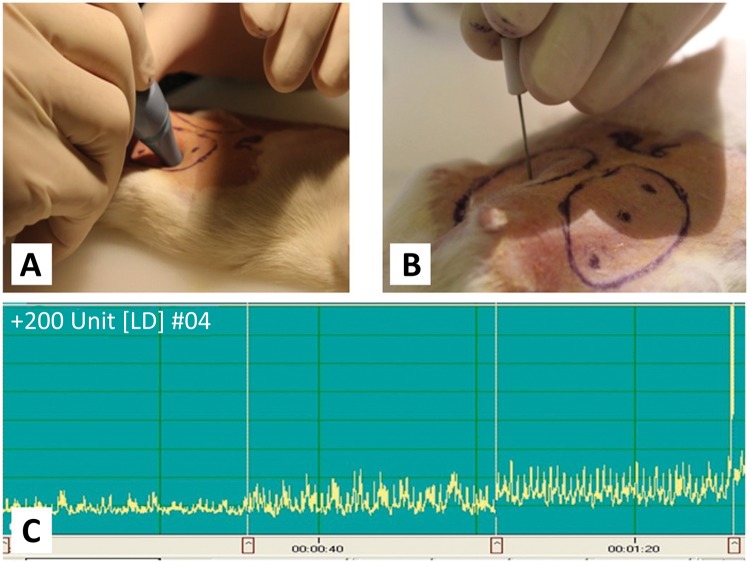
Using infrared thermometer (A) and laser Doppler pencil probe (B) taken on the defined sites of flaps. C: representative laser Doppler recording of the measurement sites (from left to right: distal, central, proximal).

Skin temperature was measured using an infrared thermometer ( Fig. 2A ). A Laser Doppler (LD) fluxmetry (LD-01 Laser Doppler Tissue Flowmeter Experimetria Ltd., Hungary) with a standard pencil probe (MNP100XP, Oxford Optronix Ltd., UK) was used for monitoring skin microcirculation ( Fig. 2B ). The device provides a relative microcirculatory parameter, the so-called Blood Flux Unit (BFU), which is an integral over the velocity and number of moving red blood cells in the examined tissue territory (cca. 1 mm^3^)^18^ . After stabilizing the recordings, 10-20-second periods (without artefacts) were analysed giving average BFU values ( Fig. 2C )^22^ .

### Histopathological examination

At the end of the 2-week follow-up period, the rats were anesthetized and the flaps were excised for histology. The excision area included the flap, the suture line and a 2-mm wide intact skin region as well. The excised tissue samples were fixed in 10% formaldehyde and sent to the Department of Pathology, where they were embedded in paraffin, and 4-µm serial sections were performed. Hematoxylin and eosin (H & E) staining were used.

### Statistical analysis

Data are presented as means ± S.D. values. One-way and repeated measures ANOVA with Bonferroni or Dunn’s post hoc tests (depending on the normality of the data distribution) were used to analyze intra- and inter-group differences. The significance level was set at p<0.05.

## Results

### Skin temperature

**Figure 3:**
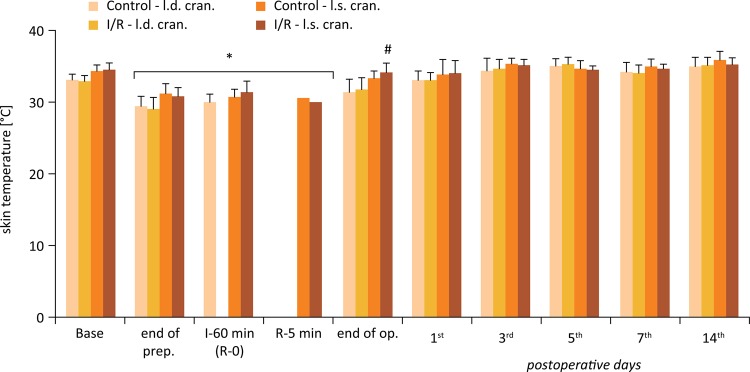
Alterations of skin temperature of the right and left cranial region of the flaps (°C) in the Control and Ischemia-Reperfusion (I/R) groups. Base = before operation; I-60 = the end of the 60-minute ischemia; R-5 = the 5th minute of the reperfusion; Means ± S.D.; *p<0.05 *vs* . Base; # p<0.05 *vs* . Control.

Skin temperature was slightly decreased on the flaps during the intra-operative period. There was no significant difference when comparing cranial, central and caudal regions of the flaps. Flaps’ skin temperature quickly recovered after repositioning. In the I/R group, local temperature (cranial region) was significantly higher by the end of the operation, when the flaps were sutured ( Fig. 3 ).

### Microcirculation 

**Figure 4:**
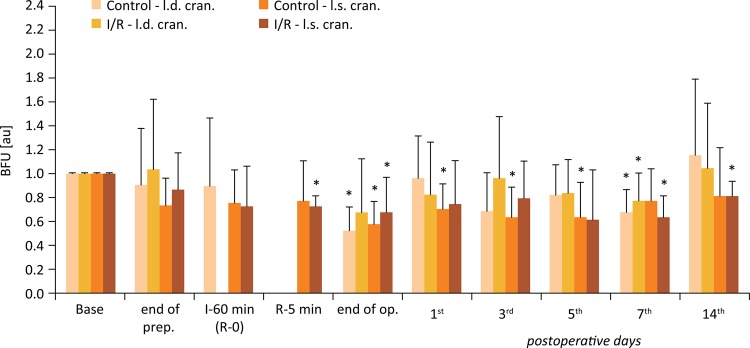
Changes of blood flux unit (BFU) relative values versus base measured on the cranial region of the flaps in the Control and I/R groups. Base = before operation; I-60 = the end of the 60-minute ischemia; R-5 = the 5th minute of the reperfusion. Means ± S.D.; *p<0.05 *vs* . Base.

**Figure 5:**
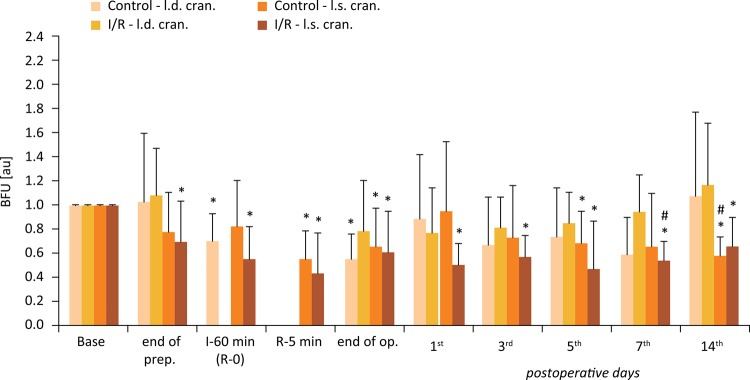
Changes of blood flux unit (BFU) relative values versus base measured on the central region of the flaps in the Control and I/R groups. Base = before operation; I-60 = the end of the 60-minute ischemia; R-5 = the 5th minute of the reperfusion. Means ± S.D.; *p<0.05 *vs* . Base; # p<0.05 *vs* . Control.

**Figure 6:**
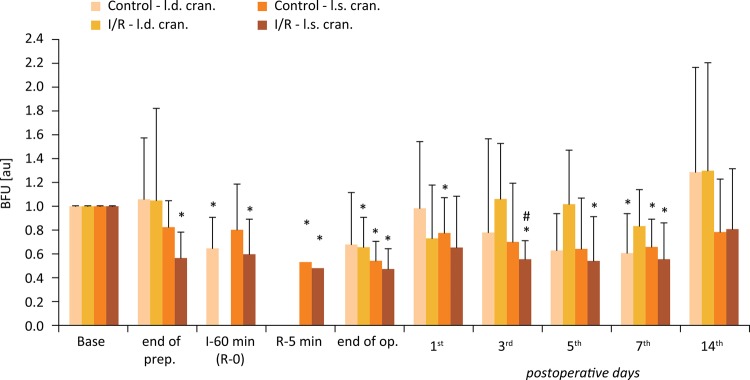
Changes of blood flux unit (BFU) relative values versus base measured on the caudal region of the flaps in the Control and I/R groups. Base = before operation; I-60 = the end of the 60-minute ischemia; R-5 = the 5th minute of the reperfusion. Means ± S.D.; *p<0.05 *vs* . Base; # p<0.05 *vs* . Control.

Figures 4 to 6 show the BFU values relative to their base. In all flaps BFU values decreased by the end of preparation, and while the flaps were elevated and hanged only on the pedicles. The magnitude of alterations was larger in the cranial region of the flaps. After ischemia, during the early minutes of the reperfusion values were still decreased. Lower values were detected by the end of the operation as well, when the sutures were finished. These values were significantly lower compared to the base values in both groups and in all flap regions. During the early postoperative days BFU values of I/R group were lower compared to the Control group. These differences were significant on the 3^rd^ day in the cranial flap region (p=0.016), on the 7^th^ and 14^th^ days in the central flap region (p=0.035 and p=0.045, respectively), and closed to the significance level on the 5^th^ day (central region, p=0.061).

### Histological findings

**Figure 7:**
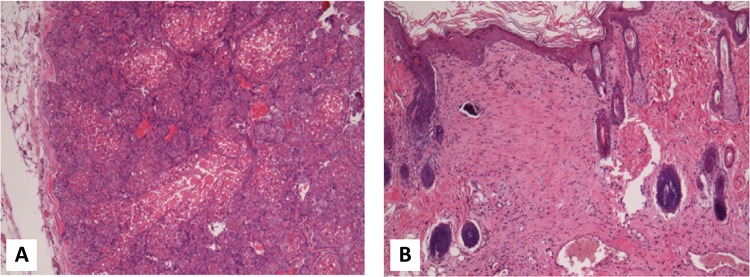
Histology of the I/R-damaged flaps. A: Hypertrophized subcutaneous mammillary glands. B: Site of the skin incision. Staining: H&E, M: 100x.

Histologically normal wound healing could be seen with granulation tissue formation at the suture line. In some flaps of the I/R group, hypertrophied inguinal mammary glands were found in the subcutaneous region ( Fig. 7 ).

### A case with complication

**Figure 8:**
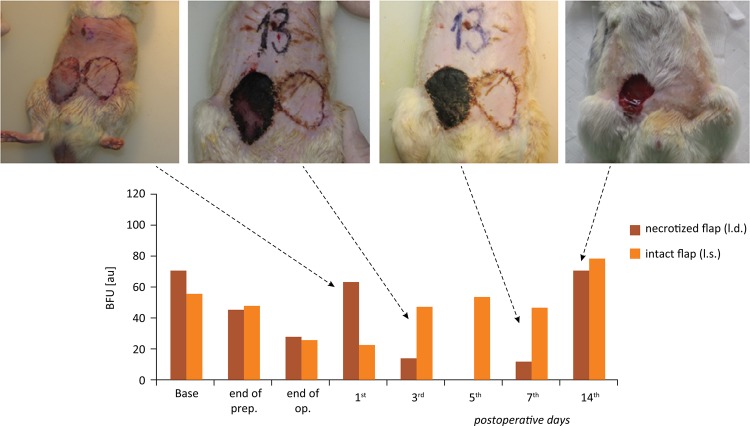
Rat #13 showed wound healing complication, with abnormal changes in skin blood flux unit (BFU) during the pre-, intra- and post- operative measurements ( *dashed arrows* shows the post-operative days and the abnormal flap).

Rat number 13 of the Control group showed flap necrosis. The individual microcirculatory data were retrospectively analyzed in this case. The values of the necrotic and healing flap were compared to the contralateral intact flap. The decrease of BFU was more prominent by the end of the operation. By the 1^st^ day a rise in BFU was seen, the flap was swollen with congestion as well. The contralateral flap remained intact. On the 3^rd^ and 7^th^ days the flap necrosis was macroscopically obvious. By the 14^th^ day BFU values normalized ( Fig. 8 ).

## Discussion

Rats are widely used in microsurgery training and research, including plastic and reconstructive surgery^13 , 14 , 26 , 27^ . By its nature, animal models still are necessary in surgical pathophysiology research, but they also have limitations when extrapolating results to humans. Epigastric adipocutaneous flap is an easy model; however, the rat vasculature shows similarities and differences as well, compared to human^15 , 28 - 31^ . In rats the angiosome of the inguinal region is supplied by the superficial inferior epigastric vessels. These vessels anatomize with the thoracoepigastric vessels and the perforator branches of inferior epigastric vessel through several connections in the central region of the abdomen. Once the inguinal skin flap was dissected and prepared, all of these anastomoses were cut and the circulation of the skin flap was reliant only on the superficial inferior epigastric vessels ( Fig. 1B ).

During the ischemic period several changes take an effect on the skin flap, such as endothelial damage, capillary thrombosis, among others^2 , 29 , 32 , 33^ . All can affect the wound healing of the flap. Collagenisation is the key component in the strength of the wound. During the first 4 weeks of the wound healing, the collagenogenesis is fast, and as a result, the wound tensile strength increases rapidly^34^ . After this period the maturation or remodeling will be the main process.

Common tools for monitoring flap viability are fluorescein dye and illumination with a Wood lamp, monitoring tissue pH and transcutaneous oxygen tension, surface temperature monitoring, Doppler ultrasound, transit-time ultrasound flowmeter, laser Doppler flowmetry (monitor, scan, confocal laser-scan)^12 , 18 - 23^ . Laser Doppler technique investigates capillary flow by the Doppler-shift in the laser beam that is backscattered from moving red blood cells. Several clinical studies showed that this method has relevance in decision-making to prevent flap failure. In this aspect the intra- or early postoperative monitoring has a great importance^1 , 3 , 20^ . In previous animal models the method was found to be useful to reflect or even predict postoperative complications^4^ .

Ischemia-reperfusion damage of flaps and complications are accompanied by further changes in several parameters, being affected by the inflammatory processes and thrombotic events. In the other study part, Magyar et al. concluded that the operation and the wound healing affected micro-rheological parameters in the early postoperative period. In I/R-damaged flaps the alterations were exacerbated on the 3^rd^-5^th^ postoperative days. In the complicated case of flap necrosis these parameters were markedly worsened^35^ . However, one of the obvious limitations of this study is the short ischemic time. Skin has a wide ischemic tolerance. Experimental models are known using much longer ischemic time, such as 4 to 8 hours^10^ . It is important to mention that the ischemic tolerance of tissues differs. In this groin flap the vasculature, its endothelial layer has a shorter ischemic tolerance^24 , 25^ . In our model detectable differences were found in laser Doppler data, suggesting that even a 1-hour ischemia and the reperfusion may cause alterations in flap perfusion.

Furthermore, the histological alterations that were seen in inguinal mammary glands in the subcutaneous fat layer also underline the importance of time factor and the necessity of reducing ischemic damage and preventing postoperative complications. In this experiment we used male rats. Compared to females, their mammary glands are dominantly lobuloalveolar with lower duct number but higher alveolus number, with frequent ductal and alveolar epithelial apoptosis^36 , 37^ . It is known that the effect of hypoxia in cells and tissues is dominantly modulated by the hypoxia-inducible factor 1 (HIF-1). Under normoxia the alpha unit of this heterodimeric transcription factor is degraded, while in hypoxia stabilizes HIF-1α. In mammary glands, the HIF-1α stimulates glucose uptake (via GLUT1 mechanism), anaerobic glycolysis, angiogenesis (by enhancing VEGF expression), and mammary development and lactation as well^38 , 39^ .

## Conclusions

Short-term ischemia could influence flap microcirculation and wound healing, and may result in hypertrophied mammary glands in this rat model. Evaluating intra- and postoperative microcirculatory changes may have significance in predicting complications. Our findings suggest that Laser Doppler fluxmetry can be used in the clinical practice, as it can predict complications before the obvious macroscopic signs occur. Using it as an additive tool can help the surgeon to intervene before flap complications arise.
